# The Effects of Ethanol and Rutin on the Structure and Gel Properties of Whey Protein Isolate and Related Mechanisms

**DOI:** 10.3390/foods11213480

**Published:** 2022-11-02

**Authors:** Na Jia, Shiwen Lin, Yuzhen Yu, Guangyao Zhang, Lingli Li, Duoduo Zheng, Dengyong Liu

**Affiliations:** National & Local Joint Engineering Research Center of Storage, College of Food Science and Technology, Bohai University, Jinzhou 121013, China

**Keywords:** whey protein isolate, rutin, ethanol, gel properties, interactions

## Abstract

The effects of different levels of rutin (0, 0.05%, 0.1%, 0.2% and 0.3% *w*/*v*) and ethanol on the structure and gel properties of whey protein isolate (WPI) were examined. The results showed that the addition of ethanol promoted the gel formation of WPI. The addition of rutin increased the gel strength of WPI and maintained the water-holding capacity of the gel. Ethanol caused an increase in thiol content and surface hydrophobicity, but rutin decreased the thiol content and surface hydrophobicity of WPI. The particle size, viscosity and viscoelasticity of WPI increased at rutin levels of 0.2% and 0.3%, indicating that rutin caused cross-linking and aggregation of WPI, but rutin had no significant effect on the zeta-potential, indicating that electrostatic interactions were not the main force causing the changes in protein conformation and gel properties. Ethanol and rutin improved the gel properties of WPI possibly by inducing cross-linking of WPIs via hydrophobic and covalent interactions.

## 1. Introduction

Whey protein isolate (WPI) is widely used in various kinds of traditional and novel foods as a valuable food ingredient because it possesses a high nutritional value and diverse functionality. One of the important properties of WPI is its gel-forming properties. Due to its gel-forming ability, WPI can be used for improving the textural properties of different food products, such as improving the viscoelasticity and chewiness of meat and dairy products. The formation of a WPI-based gel will undergo the process of “denaturation–aggregation–gelation” [1]; therefore, the factors that cause protein denaturation or aggregation will eventually affect the gel-forming properties of WPI, such as the temperature, pH, salt concentration, protein concentration and interactions with other food ingredients [1,2]. In recent years, many different methods, including changing pH, ultrasound and high-pressure treatment as well as the incorporation of ethanol, plant polyphenols and polysaccharides, have been used to improve the gel properties of WPI since these methods may modify the physicochemical structure of WPI [3,4,5].

It has been shown that ethanol can effectively lead to the denaturation and aggregation of WPI, thus affecting its gel properties [3]. Ethanol is less polar and has a lower dielectric constant than water, which makes it easy to break non-covalent interactions (such as hydrogen bonds or hydrophobic interactions), thus promoting protein denaturation and aggregation [3]. In recent years, the effects of different concentrations of ethanol ranging from 20% to 80% on the structure and gel properties of WPI have been studied [1,3,6,7]. Most studies have shown that ethanol induced significant denaturation of WPI at levels above 20%, and the best functionality was obtained at approximately 50% ethanol. However, the ethanol levels used in these studies were high, limiting the application of whey proteins in food products. Therefore, it is important to identify relatively lower levels of ethanol that are needed to promote the formation of WPI-based gel and make it possible to be used in foods that allow the use of a low content of ethanol. In future, more studies should be conducted to lower or remove the low content of ethanol after formation of a better gel.

Plant polyphenols are widespread in nature, and it is commonly known that polyphenols possess a variety of functionalities, including antioxidant activity. When incorporated into food systems, polyphenols not only act as antioxidants but also enhance the nutritional properties of foods [8]. Polyphenols are highly reactive and easily interact with the active part of protein molecules to form complexes, thus affecting the structural and functional properties of the protein [4,9]. It has been reported that anthocyanins, gallic acid, chlorogenic acid and epigallocatechin gallate lead to significant improvements in the emulsification properties of WPI [4,10,11]. Zhong et al. [12] found that the rheological properties of WPI-puerarin hydrogels could be changed by different levels of polyphenol; in particular, the complex shear modulus and hardness of the hydrogels increased when the puerarin content increased. These findings suggested the possible application of polyphenols in enhancing the functional properties of WPI.

Rutin belongs to the group of flavonoid glycosides that is widespread in asparagus, orange, grapefruit and other plants. It has been shown that the chemical structure of rutin is rich in functional groups, which can chelate with metal ions into a stable structure and exert stable biological activity, with pharmacological effects, such as anti-free radical, anti-inflammatory and anti-lipid peroxidation [13]. In recent years, rutin has received increasing attention in medical, food and nutrition research due to its wide range of biological activities. However, the effects of rutin on the gelation properties of WPI has rarely been studied. Moreover, it was reported that different concentrations of rutin had significant effects on the structure and gel properties of myofibrillar proteins, and slightly higher concentrations of rutin could ameliorate the gel properties of myofibrillar proteins [14]. It was also shown that rutin was able to increase the strength of cod skin gelatin gels by covalent interactions and produced maximum cross-linking [15]. Therefore, rutin is also anticipated to enhance the gel properties of WPI in the present study. First, different levels of ethanol were incorporated into WPI to determine the lowest ethanol level that could induce the formation of a WPI-based gel at a certain WPI level, then, different levels of rutin were added to the WPI-ethanol solution to investigate the effects of rutin on the structure and gel properties of WPI. This research aimed to clarify the structural modification of WPI by ethanol and rutin and the related changes in gel properties.

## 2. Materials and Methods

### 2.1. Materials

Whey protein isolate powder (87% protein) was obtained from Hilmar, CA, USA; ethanol (analytical purity) was obtained from Liaoning Quanrui Reagent Co, Jinzhou, China; the purity of rutin was 95% and was purchased from Shanghai Aladdin Biochemical Technology Co, Shanghai, China. All other chemical reagents used were obtained from Solarbio, Beijing, China.

### 2.2. Sample Preparation

#### 2.2.1. Preparation of Mixed Solutions of WPI and Rutin-Ethanol

WPI was dispersed into deionized water and stirred until it was completely dissolved to obtain WPI solution and then placed at 4 °C for 12 h to allow full hydration. Ethanol was mixed with the WPI solution, the pH was adjusted to 7 with 0.5 mol/L NaOH, and the reaction was carried out for 2 h at room temperature. The final concentrations of WPI in the mixed system were 7%, 7.5%, 8%, 8.5%, 9%, 9.5% and 10% (*w*/*v*), and the final levels of ethanol were 10%, 15% and 20% (*w*/*v*), respectively. To study the effects of rutin on the WPI structure and gel properties, mixtures of 8% WPI (*w*/*v*) with 15% ethanol (*w*/*v*) were added to 0.05%, 0.1%, 0.2% and 0.3% rutin (*w*/*v*). The WPI solution without ethanol and rutin was used as a control.

#### 2.2.2. Preparation of Heat-Induced Gels

The ethanol-rutin-WPI mixture was placed in a 50 mL centrifuge tube and heated in a 90 °C water bath for 30 min. The prepared gels were kept in the refrigerator (4 °C) for 12 h. The gel needed to be equilibrated at about 25 °C for 30 min before performing the gel test.

### 2.3. Total Thiol Content and Surface Hydrophobicity

The total thiol content of the proteins was measured using the Ellman reagent method according to the method of Simplicio, Cheeseman, and Slater [16]. A total of 10 mL of Tris-glycine buffer and 2 mL of protein solution were placed into a plastic centrifuge tube, mixed well and centrifuged for 15 min (4 °C, 10,000 r/min). Then, 4.5 mL of supernatant was added to 0.5 mL of 5,5′-dithiobis-(2-nitrobenzoic acid) (DTNB) solution and the reaction was carried out for 30 min and protected from light. The absorbance values were measured at 412 nm using a UV spectrophotometer (L5S; Yidian, Shanghai, China).

The surface hydrophobicity of WPI samples was determined by the method of Alizadeh and Li using 1-aniline-8-naphthalenesulfonate (ANS) as a fluorescent probe [17]. The protein concentration was diluted to 0.1–0.5 mg/mL with 0.1 mol/L sodium phosphate buffer, respectively. After that, 4 mL of WPI solution and 20 μL of 8 mmol ANS mixture were taken separately and the reaction was avoided for 10 min. The surface hydrophobicity was obtained using a fluorescence spectrophotometer (970 CRT; Jingke, Shanghai, China). The excitation wavelength was selected as 380 nm and the emission wavelength was 470 nm, the slit width was adjusted to 5 nm, and the scanning speed was adjusted to 240 nm/min.

### 2.4. Secondary Structure

Raman spectra were measured by confocal Raman spectrometer (LabRAM HR Evolution, Paris, France) to obtain Raman spectra in the analysis range of 800–1800 cm^−1^. The test parameters were a slit of 200 μm, grating of 600 g·mm^−1^, integration time of 30 s, resolution of 2 cm^−1^ and data acquisition speed of 120 cm^−1^ min^−1^.

### 2.5. Particle Size and Viscosity

A laser particle size analyzer (BT-9300ST; Baite, Dandong, China) was used to measure the particle size of WPI; water was used as the measurement medium, and the refractive index was 1.333 and the refractive index of the substance was 1.520.

The viscosity of the samples was measured using a rheometer with a rotor of 40 mm diameter, temperature adjusted to 25 °C and a shear rate of 0.1–1024 s^−1^.

### 2.6. Zeta Potential

A zeta potential meter (T Nano ZS-90; Malvern, UK) was used to measure the electrostatic interactions. The scattering angle was selected as 90° during the measurement, the equilibration time was adjusted to 60 s and the test temperature was selected as 25 °C.

### 2.7. Gel Strength, Water Holding Capacity (WHC) and Dynamic Rheology

Gel strength was measured using a TA-XT Plus Texture Analyzer (Stable Micro Systems, Godalming, UK), selecting a probe with P/0.5, measured by downward pressure with a downward pressure distance of 1/2 the gel height, a pre-test speed of 1 mm/s, a speed of 2 mm/s during the test and a post-test speed that was the same as the pre-test speed and a trigger force of 5 g.

The method of Salvador, Saguer, and Carretero was used to measure the WHC of the gel with appropriate modifications [18]. The weight of the empty centrifuge tube was indicated by *M*_0_. Approximately 10 g of gel was placed into a centrifuge tube and the weight was marked as *M*_1_. The centrifuge tube containing the gel was centrifuged for 20 min (4 °C, 5000× *g*), and then the water on the surface of the gel was lightly wiped after centrifugation, and the weight of the tube and gel at this point was indicated as *M_2_*. The WHC (%) was calculated as (*M*_2_ − *M*_0_)/(*M*_1_ − *M*_0_) × 100%.

The dynamic rheological properties of WPI were performed using a rheometer (Discovery DHR-1 Rheometer; TA, New Castle, DE, USA) equipped with serrated plate-plate geometry (40 mm diameters) and a gap between parallel plates 1 mm. The prepared protein solution was applied to the test platform to drive out the air bubbles. The rheometer frequency was adjusted to 1 Hz with a 1 mm gap between plates; 2% strain was selected; and the WPI samples were warmed from an initial temperature of 25 °C at a ramping rate of 2 °C/min to a final temperature of 95 °C. Before temperature ramp sweep tests were conducted, a preliminary amplitude sweeps tests at different temperature points (e.g., 25, 35, 45, 55, 65, 75, 85 and 95 °C). To avoid contact between the protein and air, a protective cover was placed over the solution and sealed with paraffin wax during the assay. The measurement indices were the storage modulus (G′) and loss modulus (G″).

### 2.8. Statistical Analysis

Each test was repeated three times, and the results were expressed as the mean ± standard deviation (X ± SD). Statistical analysis of data was processed using the Linear Models program in SPSS software (Version 19.0, International Business Machines Corporation, New York, NY, USA), and the LSD procedure was used for the analysis of significant differences (*p* < 0.05). The graphics were drawn using the SigmaPlot software (Version 12.5, Systat Software Inc, SAN Jose, CA, USA).

## 3. Results and Discussion

### 3.1. Photographs of WPI-Based Gels

**Figure 1:**
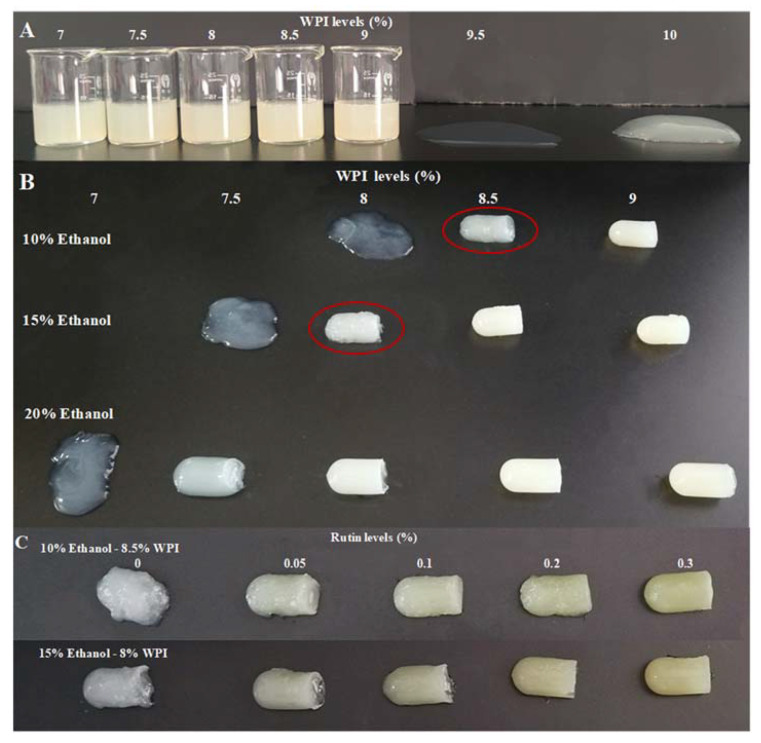
The photographs of WPI-based gels at different WPI levels (7%, 7.5%, 8%, 8.5%, 9%, 9.5% and 10% *w*/*v*) (**A**), ethanol levels (10%, 15% and 20% *w*/*v*) (**B**) and rutin levels (0.05%, 0.1%, 0.2% and 0.3% *w*/*v*) (**C**).

The effects of different WPI levels (7%, 7.5%, 8%, 8.5%, 9%, 9.5% and 10% *w*/*v*), ethanol levels (10%, 15% and 20% *w*/*v*) and rutin levels (0.05%, 0.1%, 0.2% and 0.3% *w*/*v*) on the formation of WPI-based gel were studied. Photographs of the WPI-based gels are shown in Figure 1. As shown in Figure 1A, the lowest WPI level that could form a gel was 10%, but the gel was weak, soft and shapeless. However, as shown in Figure 1B, after the addition of 10%, 15% and 20% ethanol, gels could be formed at lower WPI levels of 8.5%, 8% and 7.5%, respectively, and the gels looked harder and more shaped even at these lower WPI levels. This result indicated that ethanol promoted the formation of the WPI-based gel and lowered the WPI levels needed to form the gel. As shown in Figure 1C, after a further addition of rutin, the gels consisting of 8.5% WPI with 10% ethanol and 8% WPI with 15% ethanol became harder, smoother and more shaped than the gels with ethanol alone. These results suggested that rutin further facilitated the formation of WPI-based gel on the basis of ethanol. In the following research, 8% WPI with 15% ethanol and different levels of rutin were chosen to further investigate the changes in protein structure and gel properties induced by both ethanol and rutin to explore the possible mechanisms by which ethanol and rutin improve the gel-forming ability of WPI.

### 3.2. Changes in WPI Structure

#### 3.2.1. Total Thiol Content

**Figure 2:**
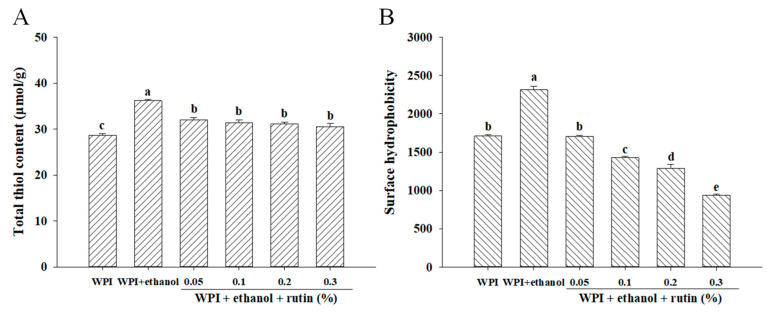
The effects of ethanol (15% *v*/*v*) and different levels of rutin (0.05%, 0.1%, 0.2% and 0.3% *w*/*v*) on the total thiol content (**A**) and surface hydrophobicity (**B**) of WPI. Means with different letters (a–e) differ significantly (*p* < 0.05).

The thiol content was measured in the present research to reveal the conformational changes and denaturation of WPI induced by both ethanol and rutin. The thiol groups in proteins have high chemical activity and are prone to be involved in various chemical reactions in food systems. It has been reported that thiol groups readily combine with phenolic compounds and their interactions in turn affect the functional properties of proteins and the texture of foods [19]. As exhibited in Figure 2A, the addition of 15% ethanol significantly (*p* < 0.05) increased the thiol content compared to the control WPI without the addition of ethanol and rutin, which may be due to unfolding of the WPI molecular structure and the exposure of thiol groups caused by ethanol [20]. It was reported that aqueous ethanol limited thiol-disulfide exchange reactions and thiol oxidation [21]; similarly, this development could also occur in the current research; therefore, the addition of ethanol increased the thiol content of WPI. WPI samples treated with different levels of rutin had significantly (*p* < 0.05) lower thiol content than the sample with 15% ethanol alone, but the difference between the different levels of rutin was not significant (*p* > 0.05). It had been reported that the decrease in the thiol content of proteins may be due to the interaction of highly reactive hydroxyl groups in phenolic compounds with the thiol groups in proteins through weak bonds, such as hydrogen bonds [22]; thus, the formation of hydrogen bonds between rutin and WPI may result in the reduction of thiol content in the present research. In addition, rutin was probably oxidized to the corresponding quinone, which could react directly with the thiol groups of WPI to form thiol–quinone adducts [19,23]. Especially at neutral pH in the present research, the thiol groups were readily deprotonated to form thiol ions (RS^−^), so they may be more prone to react with phenolic-derived quinone carbonyls and promote the formation of thiol–quinone adducts and the reduction of thiol content. It was also reported that the formation of quinones could facilitate the conversion of thiol groups to disulfide bonds, resulting in a decrease in thiol content [19,24]. Similarly, other polyphenols, such as gallic acid, chlorogenic acid and epigallocatechin gallate, were found to result in a decrease in the total thiol content of whey protein [4,24,25].

#### 3.2.2. Surface Hydrophobicity

ANS is widely used as a hydrophobic fluorescent probe to characterize hydrophobic sites on protein surfaces and to study changes in protein conformation. In general, polar amino acids are distributed on the surfaces of proteins, and nonpolar amino acids are inside the protein molecule, forming a hydrophobic core. When subjected to external forces, the protein molecular structure unfolds or folds and hydrophobic amino acids are exposed on the surface or encapsulated inside the protein molecule, thus altering the hydrophobicity of the protein [26]. As exhibited in Figure 2B, the addition of 15% ethanol significantly (*p* < 0.05) increased the surface hydrophobicity of WPI compared to the control without ethanol and rutin, because ethanol caused the unfolding of the WPI structure and exposure of the hydrophobic groups initially buried inside the protein structure, thus increasing the surface hydrophobicity. Feng et al. [3] also showed that the addition of ethanol caused higher surface hydrophobicity than natural WPI and the surface hydrophobicity increased in a dose-dependent manner. As shown in Figure 2B, the surface hydrophobicity of WPI decreased significantly (*p* < 0.05) with increasing rutin levels in a dose-dependent manner. On the one hand, the decrease in surface hydrophobicity may have been caused by the hydrophobic interactions between WPIs, which could be facilitated by previous exposure of hydrophobic groups induced by ethanol. On the other hand, noncovalent hydrophobic interactions between WPI and rutin may also occur, resulting in the introduction of hydrophilic groups of rutin binding to WPI, leading to a decrease in the hydrophobicity of the protein surface [19,27]. Furthermore, the hydrophobic interactions between WPIs or between WPI and rutin may block the ANS binding to the hydrophobic domains of WPI and reduce the hydrophobicity. Similarly, Meng et al. [4] found that the surface hydrophobicity of whey proteins decreased after binding to different polyphenols, such as gallic acid, chlorogenic acid and gallocatechin gallate. Zhang et al. [28] found that the addition of chlorogenic acid may cause polar groups (carboxyl and hydroxyl groups) of polyphenols to occupy the ANS and protein binding sites, thus reducing the surface hydrophobicity of β-lactoglobulin. As a result, in the present research, the hydrophobic interactions between WPIs or WPI and rutin may result in cross-linking and the aggregation of WPI, as confirmed by the increased particle size and viscosity as described in Section 3.3, and the cross-linking and aggregation of WPI may cause the formation of entanglements of proteins and in turn bury some hydrophobic residues inside.

#### 3.2.3. Secondary Structure

**Figure 3:**
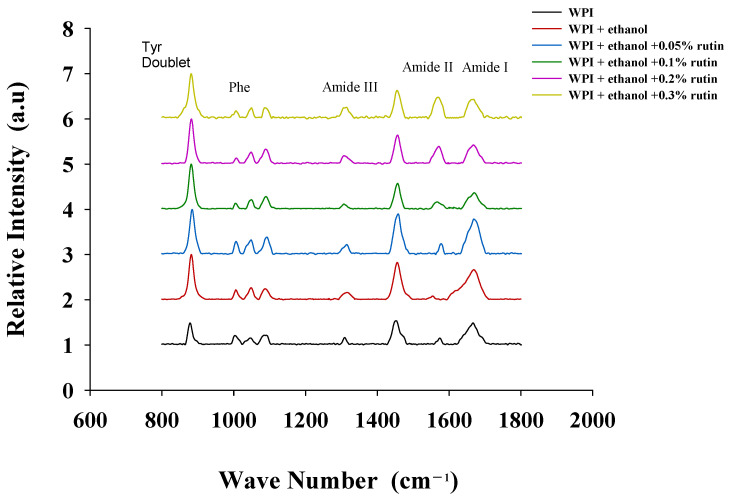
The effects of ethanol (15% *v*/*v*) and different levels of rutin (0.05%, 0.1%, 0.2% and 0.3% *w*/*v*) on the Raman spectrograms of WPI.

In general, Raman spectra reflect the changes in the secondary structure of proteins, and these changes are usually manifested in the amide I (1600–1690 cm^−1^) and amide II (1480–1575 cm^−1^) bands of the spectrum [29]. The effects of ethanol and rutin on the Raman spectrum of WPI is shown in Figure 3. Compared with the natural WPI, ethanol increased the peak of the amide I band of WPI, while the peak of amide II band almost disappeared; the addition of 0.05% rutin increased the peak of both the amide I and amide II bands, followed by decrease in the peak of the amide I band and a continued increase in the peak of the amide II band with the increased rutin levels. The C=O stretching vibrations of the peptide backbone in the amide I band can provide data on different secondary structures (α-helical, β-folded, etc.) [22].

As shown in Table 1, the addition of ethanol significantly (*p* < 0.05) decreased α-helix content and increased β-folding, β-turning and irregular coiling content. Hydrogen bonds formed by the carbonyl oxygen and amino hydrogen of protein polypeptide chains maintain the α-helix structure of protein [30], so ethanol may decrease the α-helix content by reducing the ability of WPI to form hydrogen bonds between polypeptide chains. It was confirmed that the conversion of α-helix to β-fold is beneficial for the gelation process [31]. This also explained the reason that WPI formed better gels after the addition of 15% ethanol. The addition of 0.05% rutin resulted in further significant (*p* < 0.05) reduction of α-helix content and an increase in β-folding, β-turning and irregular curling content, probably due to the low rutin levels and the dominant effect of ethanol on WPI. When rutin was added at the levels of 0.1%, 0.2% and 0.3%, the α-helix content increased and the β-folding, β-turning angle and irregular curl content decreased significantly (*p* < 0.05), indicating that high levels of rutin promoted the formation of intermolecular hydrogen bonds in WPI. This may be due to the fact that the covalent interactions between rutin and WPI changed the local sequence of amino acids in WPI and influenced its secondary structure [32]. Although the addition of high levels of rutin increased the α-helix content, the results in Section 3.3.1 (particle size) and Section 3.3.2 (viscosity) showed that rutin caused cross-linkage and aggregation of WPI, which eventually enhanced the gel strength of WPI. Similarly, Ahmed et al. [33] observed a slight increase in the α-helix content of β-lactoglobulin with the addition of caffeic acid. Kanakis et al. [34] found that the addition of catechin, epigallocatechin and epigallocatechin gallate resulted in a conformational change in β-lactoglobulin with an increase in α-helix and β-folding content, indicating the formation of a stable protein structure.

### 3.3. Crosslinking of WPI

#### 3.3.1. Particle Size

The particle size distribution can directly reflect the changes in droplet size of WPI induced by both ethanol and rutin. D10, D50 and D90 indicate the particle size corresponding to 10%, 50% and 90% of the particle size distribution, respectively. The mode is the most common diameter of the population in the peaks. The span indicates the width of the particle size distribution. D_3,2_ is the surface area mean diameter, and D_4,3_ is the volume mean diameter. As shown in Table 2, the addition of 15% ethanol alone had no significant effect (*p* > 0.05) on any particle size parameter compared to the control WPI without the addition of ethanol and rutin. When compared to WPI with 15% ethanol alone, the samples with 0.05% and 0.1% rutin had no significant (*p* > 0.05) effects on any of the other particle size parameters except for a significant increase in span (*p* < 0.05); however, the addition of 0.2% and 0.3% rutin caused a sharp and significant (*p* < 0.05) increase in all the particle size parameters. Overall, the increase in D_4,3_, D_3,2_, D10, D50 and D90 of WPI caused by 0.2% and 0.3% rutin suggested the formation of large particles, which was confirmed by the appearance of peak 2 in a larger particle range; consequently, the span became wider. The results of the present research agreed with the results of Staszewski et al. [35] who found that the β-lactoglobulin and green tea polyphenol complex exhibited larger particles. Chen et al. [36] found that high concentrations of safflower yellow led to an increase in the particle size of WPI because of the formation of macromolecular polymers at higher concentrations. Zhang et al. [28] found that the particle size distribution of β-lactoglobulin complexes was wider after adding 50–100 μmol chlorogenic acid and that the particle size of the β-lactoglobulin–chlorogenic acid complexes increased with increasing chlorogenic acid levels. As mentioned above, the interactions between ethanol and WPI unfolded the protein structure and exposed the hydrophobic groups, which may be beneficial for the subsequent hydrophobic interactions of WPI to form hydrophobic aggregates, leading to cross-linking and aggregation of protein molecules. In addition, the possible formation of disulfide bonds may contribute to the interactions of WPI. Meanwhile, rutin might act as a cross-linker via noncovalent hydrophobic interactions and covalent interactions with WPI, such as the formation of thiol–quinone adducts, and lead to the cross-linking and aggregation of WPI. Consequently, the cross-linking and aggregation of WPI may cause the polymerization of protein and the formation of entanglements, as proven by the increases in particle size.

#### 3.3.2. Viscosity

**Figure 4:**
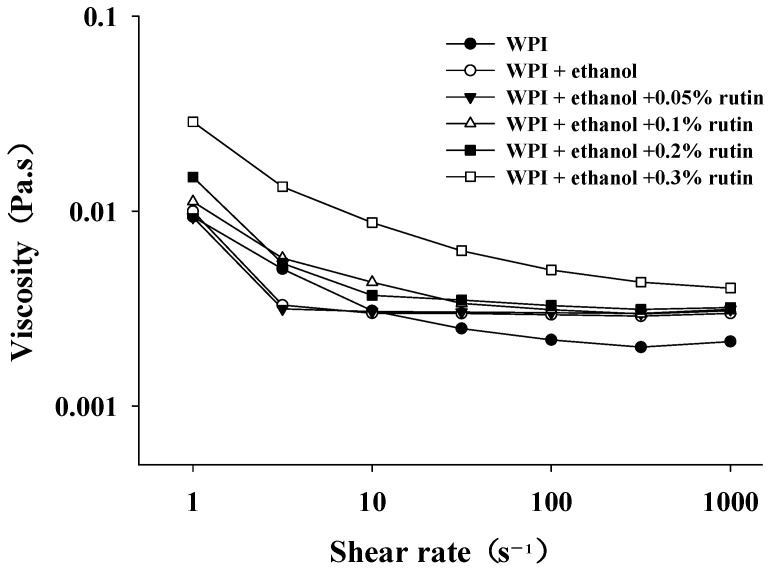
The effects of ethanol (15%, *v*/*v*) and different levels of rutin (0.05%, 0.1%, 0.2% and 0.3% *w*/*v*) on the viscosity of WPI.

Viscosity reflects the mobility of the protein and is important for applications of protein in the food industry. Usually, as the viscosity of the solution increases, the mobility of the molecules subsequently decreases. As shown in Figure 4, the viscosities of all the WPI samples decreased with increasing shear rate and exhibited non-Newtonian and shear thinning behavior. The addition of ethanol and rutin increased the viscosity of WPI, especially in the presence of 0.3% rutin. Dissanayake et al. [37] demonstrated that the viscosity of proteins was influenced by many factors, such as particle shape, particle size and size distribution, liquid polarity and surface charges of protein. For example, Coskun et al. [38] found that the viscosity increased with the increased particle size of WPI at pH 5. In the present research, the increased particle size suggested the formation of aggregates and large entanglements via the cross-linking between WPIs, which may be caused by noncovalent hydrophobic and covalent interactions. Accordingly, the formation of aggregates and large entanglements caused an increase in viscosity, further indicating that the addition of high levels of rutin gave WPI a more stable structure, so the solution still had a high viscosity under shear force. Therefore, the viscosity results confirmed that the addition of rutin caused possible cross-linking of WPI and the formation of aggregates and entanglements.

### 3.4. Zeta Potential of WPI

**Figure 5:**
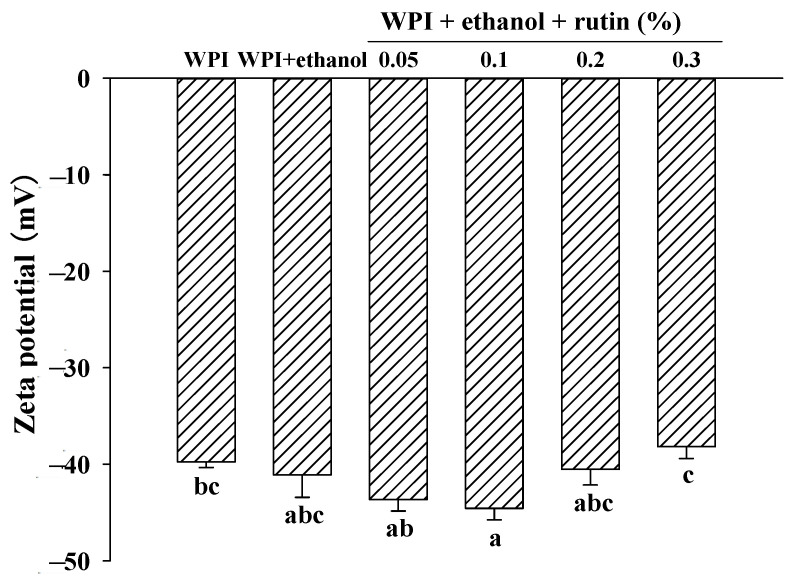
The effects of ethanol (15% *v*/*v*) and different levels of rutin (0.05%, 0.1%, 0.2% and 0.3% *w*/*v*) on the zeta-potential of WPI. Means with different letters (a–c) differ significantly (*p* < 0.05).

Zeta potential is an indicator of the stability of protein solutions by surface charge density, which can characterize the surface charge properties of proteins; the larger the absolute value of zeta potential in a certain range (−30–+30 mv), the larger the electrostatic repulsive force on the protein surface and the more stable the protein system [39,40]. As shown in Figure 5, as expected, the zeta value of the control droplets was negative because a neutral pH is greater than the isoelectric point of WPI. The addition of ethanol caused no significant (*p* > 0.05) changes in the zeta potential of WPI. When compared to WPI with 15% ethanol alone, the addition of rutin had no significant (*p* > 0.05) effect on the zeta potential of WPI; when compared to the control WPI without ethanol and rutin, 0.1% rutin caused a significant (*p* < 0.05) increase in the zeta potential of WPI, while other rutin levels had no significant (*p* > 0.05) effect on the zeta potential of WPI. At the level of 0.3% rutin, the absolute value of the zeta potential on the protein surface decreased, weakening the electrostatic repulsion between protein molecules and possibly promoting the cross-linking of protein molecules to form macromolecular aggregates, which was consistent with the results of viscosity and particle size. Xu et al. [41] also reported that the addition of resveratrol did not result in a significant change in the magnitude of the charge on the droplets. In brief, the present results suggested that electrostatic interactions may not be the main force causing the cross-linking and aggregation of WPI. Thongkaew et al. [42] found that polyphenolic compounds, including catechin, tannic acid and grape seed extract, did not significantly affect their zeta-potential values, confirming that electrostatic interactions did not play an important role in the interactions between polyphenols and proteins. Charlton et al. [43] also concluded that electrostatic interactions were not the major contributing factor to polyphenol–protein interactions.

### 3.5. Gel Strength, WHC and Dynamic Rheology

#### 3.5.1. Gel Strength and WHC

**Figure 6:**
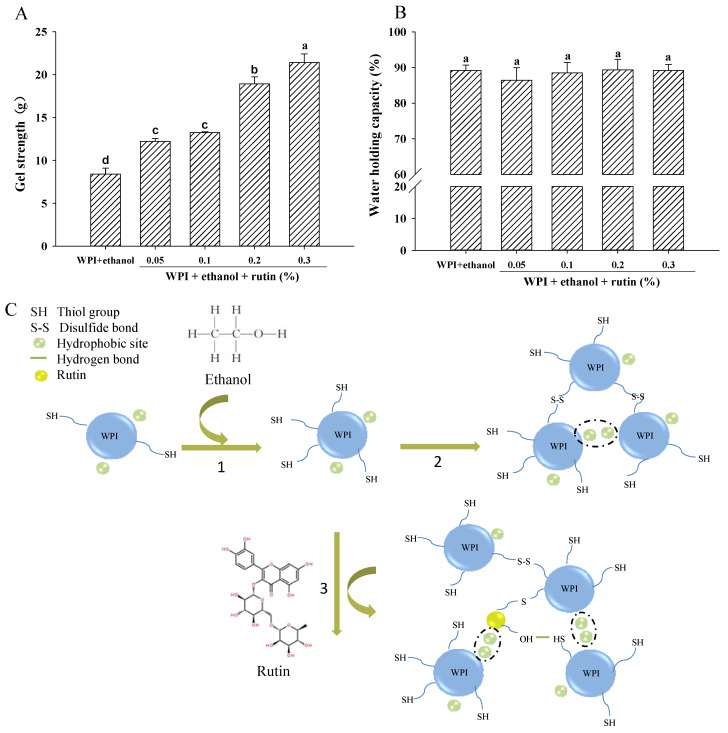
The effects of ethanol (15% *v*/*v*) and different levels of rutin (0.05%, 0.1%, 0.2% and 0.3% *w*/*v*) on the gel strength (**A**), water-holding capacity (**B**) of WPI-based gels and the proposed interactions of WPIs induced by ethanol and rutin (**C**). Means with different letters (a–d) differ significantly (*p* < 0.05). 1. Unfolding of the WPI structure and exposure of the thiol groups and hydrophobic sites of WPI; 2. Cross-linking of WPI via hydrophobic interactions and disulfide bonds induced by ethanol; 3. Rutin might act as a cross-linker via hydrophobic interactions and formation of thiol–quinone adducts and hydrogen bonds with WPI.

A sample of 8% WPI without ethanol and rutin was used as a control, but it could not form a gel; thus, the gel strength and WHC could not be measured. As shown in Figure 6A, after the addition of 15% ethanol, 8% WPI formed a gel. The results were consistent with Kleemann et al. [44] who also found that ethanol increased the WPI-based gel strength. Zirbel and Kinsella [45] reported that ethanol elevated the gel strength of WPI, possibly by enhancing electrostatic interactions or hydrogen bonding. However, as presented in Section 3.4, the addition of ethanol had no significant effect on the zeta potential of WPI, so the electrostatic interactions may not be the major contributor to the increase in gel strength in the present research. The moderate exposure of thiol and hydrophobic groups may facilitate the interactions of WPI during the heat-induced gelation process, thus increasing the gel strength. When compared to the WPI-based gel with 15% ethanol alone, the addition of rutin increased the gel strength significantly (*p* < 0.05). This may be mainly caused by the combined effects of ethanol and rutin. After adding 15% ethanol, the increased thiol content and surface hydrophobicity indicated an unfolding of the WPI structure, which may have led to more active groups participating in the gelation process and be beneficial for the formation of a gel. As mentioned earlier, the addition of rutin increased the protein viscosity and particle size, suggesting that WPI molecules may cross-link to form macromolecular aggregates before the gel structure was formed, which may contribute to the improvement of the functional properties of the gels [46]. On the one hand, the cross-linking of WPI may occur via noncovalent hydrophobic interactions and covalent disulfide bonds induced by ethanol. On the other hand, rutin might interact with WPI through hydrophobic bonds, the formation of thiol-quinone adducts and hydrogen bonds, thus it may act as a cross-linker to connect the WPI molecules, eventually resulting in the polymerization of proteins and the formation of large entanglements, which may be beneficial for the subsequent formation of heat-induced gels. The proposed interaction of WPIs induced by ethanol and rutin is shown in Figure 6C. Similarly, when the concentration of puerarin increased, the hardness of the whey protein gel increased, and the network structure of the protein became finer and more homogeneous, indicating that the addition of puerarin promoted the gelation of whey protein [12]. Staszewski et al. [34] also found that the addition of green tea polyphenols to β-lactoglobulin promoted gelation by decreasing the gel temperature and shortening the gel-formation time.

WHC is an important characteristic of protein gels and reflects the ability of the gel to retain water. As shown in Figure 6B, there was no significant (*p* > 0.05) change in the WHC of WPI-based gels after the addition of rutin. According to Figure 6A, rutin increased the gel strength of WPI; the WHC of WPI remained at a high level after rutin addition, indicating that rutin improved the gel properties of WPI.

#### 3.5.2. Dynamic Rheology

**Figure 7:**
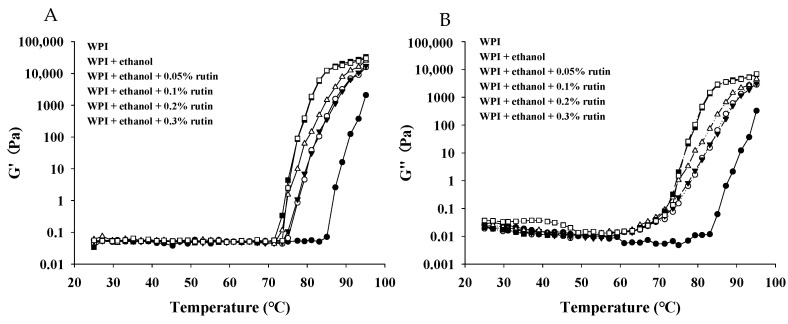
The effects of ethanol (15% *v*/*v*) and different levels of rutin (0.05%, 0.1%, 0.2% and 0.3% *w*/*v*) on the storage modulus (G′) (**A**)and loss modulus (G″) (**B**) of WPI.

Dynamic rheological experiments of temperature sweeping were conducted to observe the changes in viscoelasticity during protein gel formation. As shown in Figure 7A, the G′ values of all the WPI samples showed a flat trend from 25 °C to 75 °C; then, the G′ value of native WPI began to increase at 85 °C, indicating that the structure of WPI had changed, which was due to the aggregation of denatured WPI molecules during the heating process to form a three-dimensional gel network. After the addition of ethanol and rutin, the G′ value of WPI began to increase at approximately 75 °C, suggesting that the WPI samples more easily formed gels. When the temperature ranged from 75 °C to 95 °C, the G′ value of WPI with ethanol and rutin was higher than that of the native WPI, confirming that rutin and ethanol facilitated the gel formation of WPI and reduced the temperature required for gel formation, especially at rutin levels of 0.2% and 0.3%. As shown in Figure 7B, the G″ value of native WPI began to increase at approximately 85 °C; similar to the G′ values, after the addition of ethanol and rutin, the G″ value began to increase rapidly at approximately 70 °C. When the temperature was above 60 °C, all the WPI samples with ethanol and rutin exhibited higher G″ values than the control WPI. At the end of heating at 95 °C, the WPI samples with 0.2% and 0.3% rutin had the highest G′ and G″ values, which was consistent with the gel strength results, and the G′ values of all the samples were higher than the G″ value at a fixed test frequency, indicating that the samples formed gels with mainly elastic characteristics. Similarly, Zhong et al. [12] also found that the G′ and G″ values of whey protein increased when the concentration of puerarin was increased, suggesting that puerarin caused WPI to form stronger gels because polyphenol increased the amount or strength of the intermolecular protein bonds. Zheng et al. [47] found that WPI showed the elastic characteristics of a weak gel after the addition of epigallocatechin gallate. Harbourne et al. [48] found that the G′ value was higher than the G″ value for protein as influenced by the polyphenol (tannic acid and gallic acid) content, indicating that whey protein gels have typical elastic properties and that both the G′ and G″ values increased with increasing polyphenol concentration, which was associated with an increased degree of aggregation of the gel. In the present research, the formation of aggregates and entanglements of WPI induced by ethanol and rutin may facilitate gel formation and improve gel properties during the heating process.

## 4. Conclusions

Ethanol had a promoting effect on the gel formation of WPI so that WPI could form a gel at 8% concentration. The gel strength of WPI increased significantly after the addition of 0.2% and 0.3% rutin, and its water-holding capacity was always maintained at a high level, indicating that rutin had an improving effect on the gelation properties of WPI. The addition of rutin reduced the thiol content and surface hydrophobicity of WPI compared to that of WPI with ethanol alone, and the particle size and viscosity increased at rutin concentrations of 0.2% and 0.3%, indicating that rutin caused the cross-linked aggregation of WPI. The addition of ethanol and rutin decreased the temperature required for WPI to form gels and increased the G′ and G″. Therefore, the addition of rutin and ethanol can cause cross-linked aggregation of WPI to effectively improve the gel properties of WPI. Further research should be conducted to investigate the changes in the biofunctionality of WPI as affected by the addition of ethanol and rutin.

## Figures and Tables

**Table 1:** The effects of ethanol (15% *v*/*v*) and different levels of rutin (0.05%, 0.1%, 0.2% and 0.3% *w*/*v*) on the secondary structure of WPI. Means in the same row with different letters (a–d) differ significantly (*p* < 0.05).

Secondary Structure (%)	WPI	WPI + Ethanol	WPI + Ethanol + Rutin (%)			
			0.05	0.1	0.2	0.3
α-helix	25.06 ± 0.02 ^c^	19.79 ± 0.35 ^cd^	14.51 ± 0.46 ^d^	35.96 ± 0.01 ^b^	41.24 ± 0.01 ^b^	57.42 ± 0.45 ^a^
β-Folding	42.83 ± 0.00 ^b^	46.94 ± 0.27 ^ab^	51.05 ± 1.92 ^a^	34.34 ± 0.00 ^c^	30.23 ± 0.00 ^c^	17.63 ± 0.25 ^d^
β-turning	19.97 ± 0.01 ^a^	20.81 ± 0.05 ^a^	21.65 ± 0.40 ^a^	18.24 ± 0.02 ^b^	17.40 ± 0.00 ^b^	14.82 ± 0.87 ^c^
Irregular curl	12.14 ± 0.02 ^b^	12.46 ± 0.02 ^ab^	12.79 ± 0.16 ^a^	11.46 ± 0.00 ^c^	11.13 ± 0.00 ^c^	10.13 ± 0.34 ^d^

**Table 2:** The effects of ethanol (15% *v*/*v*) and different levels of rutin (0.05%, 0.1%, 0.2% and 0.3% *w*/*v*) on the particle size parameters of WPI. “—” means peak2 is not detected. Means in the same row with different letters (a–d) differ significantly (*p* < 0.05).

Particle Size Parameters (μm)	WPI	WPI + Ethanol	WPI + Ethanol + Rutin (%)			
			0.05	0.1	0.2	0.3
D_4,3_	2.01 ± 0.00 ^c^	1.97 ± 0.01 ^c^	2.11 ± 0.00 ^c^	2.16 ± 0.06 ^c^	24.08 ± 0.22 ^b^	31.45 ± 0.12 ^a^
D_3,2_	1.58 ± 0.04 ^c^	1.55 ± 0.00 ^c^	1.59 ± 0.00 ^c^	1.64 ± 0.00 ^c^	4.17 ± 0.04 ^b^	5.57 ± 0.24 ^a^
D10	0.88 ± 0.01 ^d^	0.89 ± 0.00 ^cd^	0.86 ± 0.00 ^d^	0.94 ± 0.00 ^c^	1.52 ± 0.01 ^b^	2.27 ± 0.06 ^a^
D50	1.87 ± 0.05 ^c^	1.82 ± 0.00 ^c^	1.93 ± 0.00 ^c^	1.97 ± 0.00 ^c^	20.56 ± 0.22 ^b^	22.27 ± 0.39 ^a^
D90	3.34 ± 0.14 ^c^	3.29 ± 0.00 ^c^	3.65 ± 0.01 ^c^	3.74 ± 0.00 ^c^	53.38 ± 0.85 ^b^	62.14 ± 0.58 ^a^
Span	1.32 ± 0.03 ^d^	1.32 ± 0.00 ^d^	1.45 ± 0.00 ^c^	1.42 ± 0.00 ^c^	2.52 ± 0.07 ^b^	2.69 ± 0.08 ^a^
Mode Peak1	2.15 ± 0.00	2.15 ± 0.00	2.15 ± 0.00	2.15 ± 0.00	2.15 ± 0.00	2.15 ± 0.00
Peak2	—	—	—	—	30.39 ± 0.00	30.39 ± 0.00

## Data Availability

Data is contained within the article.
